# C-853-02. Cutaneous Functional Units as Predictors of Permanent Impairment After Burn Injury

**DOI:** 10.1093/jbcr/irag033.070

**Published:** 2026-04-06

**Authors:** Maya F Andrade, Rupak Mukherjee, Jenna C Kelly, Rohit Mittal, Steven A Kahn

**Affiliations:** Medical University of South Carolina, Charleston, SC; Medical University of South Carolina, Charleston, SC; South Carolina Burn Center at Medical University of South Carolina, Charleston, SC; South Carolina Burn Center at Medical University of South Carolina, Charleston, SC; Medical University of South Carolina, Charleston, SC

## Abstract

**Introduction:**

Burn injury can result in permanent impairment, significantly impacting patient function and quality of life. Many people sustain burns on the job, often necessitating a calculation of a formal impairment rating for their worker’s compensation claim. The American Medical Association’s Guides to the Evaluation of Permanent Impairment (AMA Guide) serves as the gold standard for impairment assessment. Integumentary system impairment involves symptoms, nature of the scar, impairment of activities of daily living, intensity of treatment to maintain function, and size of the lesion. Cutaneous functional units (CFUs), defined as fields of skin engaged in single joint motion, have been recently introduced as a more precise way than total body surface area (TBSA) to quantify how subareas of skin contribute to mobility, offering improved functional segmentation while preserving overall TBSA context. To date, CFUs have not been used to calculate impairment ratings, as they are not explicitly mentioned in the AMA Guide. Because scar formation following burn injury is ultimately what drives long-term functional limitations, it was hypothesized that scar burden measured by CFUs would predict impairment rating. The purpose of this study was to determine the relationship between initial burn TBSA, initial CFUs injured, final CFUs of the scar, and impairment ratings in burn patients at the time of maximum medical improvement (MMI).

**Methods:**

A retrospective review of 78 burn patients at an ABA-verified burn center was conducted. Inclusion criteria were photographically documented TBSA at injury and whole body impairment ratings at MMI determined by a single fellowship-trained burn surgeon using the current AMA Guide. Initial CFUs were calculated from burn injury photographs using a previously established CFU map, and scar CFUs were from scar photographs at impairment rating. Correlations between burn size, initial CFUs, scar CFUs, and impairment rating were assessed using Spearman’s rho.

**Results:**

Median values for initial TBSA, initial CFUs, scar CFUs, and impairment rating are summarized in Table 1. Significant correlations were observed among all these variables, with corresponding rho and *p*-values presented in Table 2. Scar CFUs at MMI had the strongest correlation with impairment rating.

**Conclusions:**

Initial burn size, initial burn CFUs, and permanent scar CFUs positively correlated with each other and final impairment ratings. Scar CFUs demonstrated the strongest relationship with final impairment. These findings support the hypothesis that CFUs are a stronger predictor of impairment ratings and suggest scar CFUs organically play an important role in permanent impairment, although not specifically part of the AMA Guide calculations.

**Applicability of Research to Practice:**

Future editions of the AMA Guide should consider integrating scar CFUs into their methodology.

**Funding for the study:**

N/A.

